# A Report on a Family with *TMTC3*-Related Syndrome and Review

**DOI:** 10.1155/2020/7163038

**Published:** 2020-11-04

**Authors:** Sayeeda Hana, Deepak karthik, Jingxuan Shan, Stephany El Hayek, Lotfi Chouchane, André Megarbane

**Affiliations:** ^1^Centre for Arab Genomic Studies, Dubai, United Arab Emirates, Qatar; ^2^Genetic Intelligence Laboratory, Weill Cornell Medicine-Qatar, Qatar Foundation, Doha, Qatar; ^3^Department of Genetic Medicine, Weill Cornell Medicine, New York, NY, USA; ^4^Department of Microbiology and Immunology, Weill Cornell Medicine, New York, NY, USA; ^5^Department of Human Genetics, Gilbert and Rose-Marie Ghagoury School of Medicine, Lebanese American University, Byblos, Lebanon; ^6^Institut Jérôme Lejeune, Paris, France; ^7^Faculty of Medical Sciences, Lebanese University, Lebanon

## Abstract

Recessive mutations in the *TMTC3* gene have been reported in thirteen patients to date exhibiting development delay, intellectual disability (ID), seizures, and muscular hypotonia, accompanied occasionally by neuronal migration defects expressed as either cobblestone lissencephaly or periventricular hypertopia. Here, we report a new case of a *TMTC3*-related syndrome in a Lebanese family with two affected siblings showing severe psychomotor retardation, intellectual disability, microcephaly, absence of speech, muscular hypotonia, and seizures. Whole exome sequencing revealed a homozygous pathogenic variant c.211 *C* > *T* (p.R71C) in the *TMTC3* gene in both siblings. A review of the literature on *TMTC3*-related syndrome and its causal mutations is provided.

## 1. Introduction

The protein O-mannosyl-transferase *TMTC3* belongs to a family of four recently discovered ER transmembrane O-mannosyl-transferases, each of which is characterized by the presence of four tetratricopeptide repeats. Recent studies have shown that this protein is involved in the O-mannosylation of cadherins and protocadherins, thereby affecting cellular adhesion [1]. In addition, there is some evidence to support the role of *TMTC3* in modulating the ER stress response [2].

Recessive mutations in the *TMTC3* gene have been reported in thirteen patients to date exhibiting development delay, intellectual disability (ID), seizures, and muscular hypotonia [3, 4]. Many of these patients show a phenotype which is accompanied by neuronal migration defects expressed as either cobblestone lissencephaly or periventricular hypertopia.

In this study, we report a Lebanese family with *TMTC3*-related syndrome with two siblings showing severe psychomotor delay, intellectual disability, microcephaly, absence of speech, muscular hypotonia, generalized seizures, and carrying a homozygous pathogenic variant in the *TMTC3* gene. We also provide a review of the literature of patients with causal mutations in this gene.

## 2. Materials and Methods

The proband and her sister, from healthy, first-cousin parents, were seen in our clinic in Beirut, Lebanon. They underwent an extensive workup including a thorough clinical evaluation, total body X-rays, echocardiography, karyotype, routine blood tests, and molecular analysis.

### 2.1. DNA Isolation

Informed consent for genetic analysis was obtained from the family in compliance with national ethics regulation. Genomic DNA for the two sisters and their parents was isolated from blood samples using standard techniques.

### 2.2. Exome Sequencing

Shotgun libraries were generated from 1 *μ*g of genomic DNA (gDNA). For each sample, gDNA was sheared to 150–200 bp using Covaris E220 instrument (Covaris, Inc.) under the following conditions: 50 *μ*l total volume, 10% duty cycle, 175 as intensity, 200 cycles per burst, and 360 seconds in the frequency sweeping mode.

The rest of the library preparation followed the manufacturer's instructions as described in the SureSelectXT2 Library Prep Kit (Agilent Technologies, Inc.). In brief, the sheared DNA was end-repaired and A-tailed to generate blunt ends and then ligated to Illumina compatible indexed adaptors; finally, the adaptor-ligated DNA fragments were PCR-enriched. The quality and quantity of each library were assessed with the 2100 Bioanalyzer DNA 1000 Assay (Agilent Technologies, Inc.), to confirm a distribution with an average DNA fragment size of approximately 250 to 275 bp. Equal amounts of each indexed whole genome library were pooled to make 1500 ng in 7 *μ*l. The pooled libraries were hybridized to SureSelect Human All-Exon V6 (Agilent Technologies, Inc.), following manufacturer's instructions. Then, the SureSelect-enriched indexed library DNA pool was PCR-amplified. Finally, the amplified captured libraries were purified using Agencourt AMPure XP beads (Beckman Coulter, Inc.). The quality of the library was assessed with the 2100 Bioanalyzer High Sensitivity DNA assay (Agilent Technologies, Inc.). Captured libraries were sequenced on a single lane on the Illumina HiSeq4000 in a paired-end 150 bp run following the manufacturer's instructions and using the standard sequencing primer.

### 2.3. Bioinformatic Analysis

The captured libraries were sequenced using the Illumina HiSeq4000 Analyzer following the manufacturer's instructions (Illumina, San Diego, USA). The reads were mapped to human genome reference (hg19) using Burrows–Wheeler Aligner (http://bio-bwa.sourceforge.net/). Single-nucleotide variants and indels were called with GATK 3.8, SAMtools (http://samtools.sourceforge.net/), and Picard (http://broadinstitute.github.io/picard/). All single-nucleotide variants and indels were filtered via multiple databases. Pathogenic variants were assessed under the protocol issued by the American College of Medical Genetics and Genomics [5].

## 3. Results

### 3.1. Case Report

**Figure 1:**
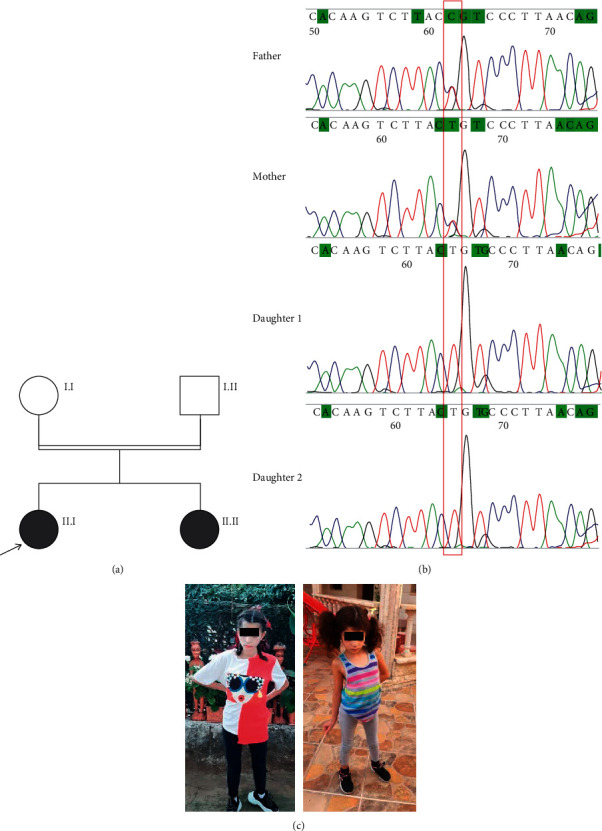
(a) Pedigree of the affected family. (b) Sanger sequencing electropherograms showing the *TMTC3* variant c.211C > T (red box) at a heterozygous state in parents and at a homozygous state in the affected daughters. (c) Photographs of the patients.

The proband (Figure 1(a), II.I) was born at term after a normal pregnancy with generalized hypotonia, a head circumference (OFC) of 33 cm (25^th^ centile); a weight of 1800 g (3^rd^ centile); and a length of 48 cm (35^th^ centile). She had a delay in developmental milestones as she sat at age 2 and started to walk at age 3.

At the age of 5 years, her parents started to note episodic crises, mainly nocturnal, consisting of abnormal breathing and generalized stiffening and contraction of the limbs. Different treatment regimens were initiated, until she was more or less stabilized under Depakine and Rivotril.

She presented to the clinic at the age of 14 years (Figure 1(c)). She had severe psychomotor delay which was speechless and could understand simple orders. She was moving continuously, had a tip-toe walk, had stereotypic hand movements, and hit herself from time to time. Her OFC was 50.8 cm (<3^rd^ centile), her height was 143 cm (<3^rd^ centile), and her weight was 33 kg (<3^rd^ centile). Upon examination, she had no dysmorphic facial features. A single palmar crease was noted on the right hand. She showed muscular hypotonia and had no osteotendinous reflexes. She had a delay in puberty as breasts had just started to grow, and she still had an absence of menses. Cardiac and ophthalmological examination, as well as brainstem auditory evoked response, was normal. Brain MRI showed the presence of an Arnold-Chiari type 1 malformation (Table 1).

Her younger sister (Figure 1(a), II.II) was born 7 years later after a normal pregnancy. At birth, OFC was 33.2 cm (25^th^ centile); weight was 2410 g (5^th^ centile); and length was 47 cm (25^th^ centile). According to the parents, she had the same clinical course as of her affected sister. Nocturnal episodes of seizures appeared at age 3.5 years and were controlled by Convulex and Lovipram.

She was seen at the age of 8 years (Figure 1(c)). Her OFC was 49.7 cm (5^th^ centile), height was 123 cm (50^th^ centile), and weight was 17 kg (<3^rd^ centile). She had severe psychomotor delay and displayed autistic behaviour. She was hypotonic, still could not walk alone, and, when helped, had a severe ataxia. However, in her case, she had a divergent strabismus, a hypodontia, a left single palmar crease, and no cardiac abnormalities (Table 1). MRI was performed but did not reveal any remarkable findings.

For both sisters, results of routine blood analyses and karyotype were all unremarkable.

### 3.2. Molecular Analysis

**Figure 2:**
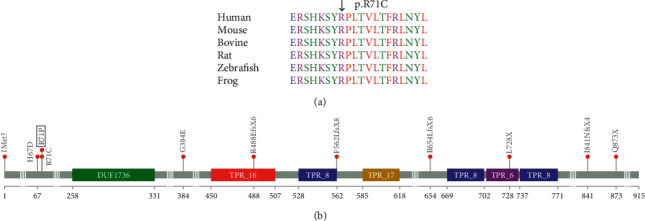
(a) ClustalW alignment of *TMTC3* residues showing conservation of the p.71R residue across diverse species. (b) Lollipop plot of the known mutations on the *TMTC3* protein.

Whole exome sequencing (WES) revealed the presence of a homozygous pathogenic variant c.211C >T (p.R71C) in the *TMTC3* gene in both affected sisters. The parents were both found to be heterozygous for this variant. The presence of this variant was confirmed by Sanger analysis (Figure 2(b)). This variant introduces a missense mutation, which is predicted to be a damaging mutation altering the structure of the protein and its biological role. The variant was not found in the 1000 Genomes Project (http://www.1000genomes.org/home) or the NHLBI Exome Sequencing Project (http://evs.gs.washington.edu/EVS/), but it was found in dbSNP (rs758540759) and the Genome Aggregation Database (gnomAD; https://gnomad.broadinstitute.org/). In the latter, the variant was extremely rare, being detected in only two out of 280532 individuals; both of whom were heterozygous. The CADD score of 32 indicates the variant is within the top 0.1% deleterious variants in the human genome [6]. SIFT function prediction was damaging, while PolyPhen-2 function prediction was probably damaging. To determine amino acid conservation, human protein sequences were aligned to the other protein sequences from a dispersed set of species using ClustalW [7], highlighting the conservation at the residue affected in the siblings reported here (Figure 2(a)).

## 4. Discussion

Here, we report a Lebanese family with two siblings affected with severe psychomotor retardation, ID, and muscular hypotonia, with homozygous mutations in the *TMTC3* gene. To date, 13 patients with homozygous or compound heterozygous mutations in this gene, from six families, have been reported [3, 4].

A comparison of phenotypes between the patients clearly marks mild-to-moderate ID and the presence of seizures as the common clinical features in all cases (Table 1). In the patients described here, psychomotor development was severely delayed, as it was in a few cases reported by Jerber et al. [3]. However, in the Pakistani family [4], no such delay was noted. Speech development has also been reported to range from normal to severe delay. The two siblings reported here had absent speech, with the older sibling still unable to speak at 14 years of age. Truncal hypotonia has also been reported in most patients, as is the case here. Based on the data available so far, we propose a clinical key for *TMTC3*-related disorders. The core phenotype for this disorder includes ID in the presence of generalized tonic-clonic, myoclonic, or nocturnal seizures. In addition, most patients may also show a combination of additional features, such as psychomotor and/or language delay, muscular hypotonia, autistic behaviour, and brain findings suggestive of neuronal migration defects.

A comparison of all the 11 *TMTC3* mutations so far known to be associated with a pathogenic phenotype showed some interesting results (Table 2). All mutations are located within the exons and are either missense, nonsense, or frameshift mutations. Splice site mutations have not so far been observed. We used lollipops, a variant visualization tool [8], to map these 11 mutations onto the primary structure of the *TMTC3* protein (Figure 2). The mutations were found to be located across several regions of the protein. The most severe reported phenotypes, especially with relation to psychomotor and speech delay, were associated with homozygous mutations towards the *N*-terminal part of the protein. Interestingly, the c.211C > T mutation we report in our patients affects the same arginine residue at codon 71 that has been shown to be mutated in one of the previously reported families [4]. Although the phenotype of the ID and seizures is similar between these patients, the latter family had a milder phenotype, especially in relation to motor and speech development. This discrepancy could be explained by the fact that, in the family reported here, the mutation was present in a homozygous state, while the patients with the milder phenotype carried compound heterozygous mutations, with the second mutation affecting the C-terminal domain.

It is known that glycosylation of cell-surface binding receptors, such as cadherins and alpha-dystroglycan, is important for maintaining the interaction between the glial limitans and the extracellular matrix. Defects in mannosylation of these proteins result in developmental disorders associated with neuronal migration defects [9]. In our study, no such migration defects were noted. An Arnold-Chiari type I malformation was visible on the first brain scan, but not in a subsequent scan 10 years later. No other anomalies were noted. This is of interest since all patients reported so far, with the exception of the youngest sibling in the Pakistani family, were reported to have migration defects in the MRI, either in the form of cobblestone lissencephaly or periventricular hypertopia.

With the exception of the one patient from the USA, all others are from the Eastern Mediterranean region. In fact, 6 of the original 13 patients are from the Arab ancestry. Rare recessive disorders tend to appear in populations with a high rate of consanguinity [10, 11]. Apart from the Pakistani family and the American patient, all other patients come from consanguineous families and carry homozygous *TMTC3* mutations.

The ease of access to high-throughput molecular technologies such as whole exome sequencing (WES) and whole genome sequencing (WGS) in the clinic has opened new vistas for the molecular diagnoses of patients with rare genetic disorders [12, 13]. Particularly for disorders that have not yet been clearly phenotypically delineated, as are many ID disorders, WES offers a straight-forward and reliable diagnostic tool, which in turn will lead to a more comprehensive clinical characterization of such disorders.

## Figures and Tables

**Table 1:** Phenotypic and genotypic features of patients reported with *TMTC3* mutations.

Family	Ethnicity	No.	Psychomotor development	Speech development	ID	Hypotonia	Brain MRI findings	Autistic behaviour	Seizure	*TMTC3* mutation	Reference
1	Egypt	1.1	Delayed	Delayed	+	+	Cobblestone lissencephaly, ventriculomegaly, encephalocele, brainstem hypoplasia, cerebellar dysplasia		GTC	c.1462delA/c.2617C > T	Jerber et al. [3]
2	Yemen	2.1	Delayed	Delayed	+	+	Cobblestone lissencephaly, ventriculomegaly, brainstem hypoplasia, cerebellar hypoplasia	+	GTC	c.1959_1960insTT	Jerber et al. [3]
		2.2	Delayed	Delayed	+	+	Cobblestone lissencephaly, ventriculomegaly, brainstem hypoplasia, cerebellar hypoplasia	+	GTC	c.1959_1960insTT	Jerber et al. [3]
3	Egypt	3.1	Delayed	No speech	+	+	Cobblestone lissencephaly, corpus callosum hypoplasia, brainstem hypoplasia, cerebellar hypoplasia, retrocerebellar cysts	−	Myoclonic	c.1686_1701del	Jerber et al. [3]
4	Lebanon	4.1	Delayed	No speech	+	+	Ventriculomegaly, corpus callosum hypoplasia, brainstem hypoplasia, cerebellar dysplasia, hypomyelination		−	c.199C > G	Jerber et al. [3]
		4.2	Delayed	No speech	+	+	Ventriculomegaly, corpus callosum hypoplasia, brainstem hypoplasia, cerebellar dysplasia, hypomyelination		−	c.199C > G	Jerber et al. [3]
5	Turkey	5.1	Delayed	Delayed	+	+	Cobblestone lissencephaly, ventriculomegaly	−	−	c.3G > A	Jerber et al. [3]
		5.2	Delayed	Delayed	+	+	Cobblestone lissencephaly	−	GTC	c.3G > A	Jerber et al. [3]
6	USA	6.1	Delayed	No speech	+	+	Cobblestone lissencephaly, ventriculomegaly, encephalocele	−	Infantile spasms	c.1151G > A/c.2521dupA	Jerber et al. [3]
7	Pakistan	7.1	−	−	+		Bilateral periventricular heterotopia	−	Nocturnal seizures	c.432G > C/c.2183T > A	Farhan et al. [4]
		7.2	−	−	+		Bilateral periventricular heterotopia, venous anomaly	−	Nocturnal seizures	c.432G > A/c.2183T > A	Farhan et al. [4]
		7.3	−	Delayed	+		Bilateral periventricular heterotopia, Arnold-Chiari type I malformation	−	Nocturnal seizures	c.432G > A/c.2183T > A	Farhan et al. [4]
		7.4	−	−	+		Normal MRI	−	Nocturnal seizures	c.432G > A/c.2183T > A	Farhan et al. [4]
8	Lebanon	8.1	Delayed	No speech	+	+	Arnold-Chiari type I malformation	+	Nocturnal seizures	c.211C > T	This study
		8.2	Delayed	No speech	+	+	Normal MRI	+	Nocturnal seizures	c.211C > T	This study

Note: +: present sign; −: absent sign; GTC: generalized tonic-clonic.

**Table 2:** Comparison of *TMTC3* mutations reported in literature.

Mutation (NM_181783.4)	Consequence	HGVSp	Exon	dbSNP	SIFT	PolyPhen	CADD PHRED
c.3 G > A	Start lost	p.Met1	2	rs1057517698	0	0.09	24
c.199 C > G	Missense	p.His67Asp	3	rs754200057	0	0.998	26.3
c.211 C > T	Missense	p.Arg71Cys	3	rs758540759	0	1	26.1
c.212 G > C	Missense	p.Arg71Pro	3	rs770896677	0	1	32
c.1151 G > A	Missense	p.Gly384Glu	8	−	0	0.999	29.3
c.1462delA	Frameshift	p.Arg488GlufsTer6	11	rs1057517696	−	−	−
c.1686_1701del	Frameshift	p.Phe562LeufsTer8	12	−	−	−	−
c.1959_1960insTT	Frameshift	p.Arg654LeufsTer6	14	rs1057519417	−	−	−
c.2183 T > A	Stop gained	p.Leu728Ter	14	−	−	−	40
c.2521dupA	Frameshift	p.Ile841AsnfsTer4	14	−	−	−	−
c.2617 C > T	Stop gained	p.Gln873Ter	14	rs1057517697	−	−	37

Mutations are arranged according to position on the protein.

## Data Availability

No data were used to support this study.
